# Delayed Flowering in Bamboo: Evidence from *Fargesia qinlingensis* in the Qinling Mountains of China

**DOI:** 10.3389/fpls.2016.00151

**Published:** 2016-02-16

**Authors:** Wei Wang, Scott B. Franklin, Zhijun Lu, Brian J. Rude

**Affiliations:** ^1^Analytic Consulting Group, EpsilonBlue Ash, OH, USA; ^2^School of Biological Sciences, University of Northern ColoradoGreeley, CO USA; ^3^Key Laboratory of Aquatic Botany and Watershed Ecology, Wuhan Botanical Garden, Chinese Academy of SciencesWuhan, China; ^4^H. W. Essig Nutrition Lab, Mississippi State UniversityStarkville, MS USA

**Keywords:** biomass, clonal, delayed flowering, energy allocation, gregarious, habitat modification, predator satiation

## Abstract

Gregarious flowering of bamboo species impacts ecosystem properties and conservation, but documentation of these periodic events is difficult. Here, we compare the characteristics of flowering sites and un-flowered patches of an arrow bamboo (*Fargesia qinlingensis*) in the Qinling Mountains, China, over a 5-year period (2003–2007) after a mast flowering event (2003). We examined flowering culm and seedling characteristics in relation to questions regarding the evolution of delayed flowering. Density of live culms decreased over the 5 years in both flowering sites and un-flowered patches. New shoots regenerated only in un-flowered patches. Chemical constituent allocation varied among culm parts (stems, branches, and leaves). Crude protein and extract ether in branches and leaves were less in flowering culms than in un-flowered culms. Seedling density was lower than expected based on floret counts, suggesting predation of seeds. Seedling density was significantly greater in flowering sites than in un-flowered patches and decreased over time. Seedlings performed better in flowering sites than in un-flowered patches based on their height, leaf number per seedling, and average leaf length, while fertilization on flowering sites had no significant effect on seedling growth, suggesting a saturation of resources. This study suggested that the characteristics of bamboos and bamboo stands were dramatically altered during this flowering event, enhancing seedling establishment and growth, and supporting mostly the habitat modification hypothesis of delayed reproduction.

## Introduction

Many bamboo species are semelparous, having an unusual life history including a long clonal growth phase (sometimes over 100 years), followed by mass synchronous flowering and subsequent death (Janzen, 1976; Tian, 1987; Qin et al., 1989; Taylor et al., 1991). The long inter-mast period has hindered documentation of stand changes (Widmer, 1998; Marchesini et al., 2009; Abe and Shibata, 2012; Austin and Marchesini, 2012) and a thorough understanding of bamboo regeneration mechanisms and patterns, because flowering events are relatively rare (but see Kakishima et al., 2011 and de Carvalho et al., 2013). Thus, the evolutionary selection for such long vegetative phases, and semelparity, in bamboos is still debated (Keeley and Bond, 1999; Saha and Howe, 2001; Franklin, 2004; Iler and Inouye, 2013). Bamboo forests also provide habitat throughout the world (Franklin, 2004; Nath and Das, 2010), but sporadic die-off events result in the loss of habitat and diversity (Sertse et al., 2011) for several years threatening endangered species like the giant panda (Tian, 1987). In addition, bamboo flowering events are linked to heterospecific tree species and thus forest regeneration patterns (Kitzberger et al., 2007; Caccia et al., 2009; Montti et al., 2014; Caccia et al., 2015). For these reasons, it is imperative to advance our understanding of the patterns and mechanisms of bamboo flowering events. In this study, we examined changes of stand structure (*habitat modification hypothesis*), seed bank dynamics and seedling regeneration (*seed predator hypothesis*), and culm characteristics/energy allocation (*resource hypothesis*) during a flowering event of *Fargesia qinlingensis* T.P. Yi and J. X. Shao in order to better understand the biology of gregarious-flowering, semelparous long-lived bamboos, and subsequently manage for habitats of endangered species following die-offs (Nath and Das, 2010).

One theory for delayed reproduction is based on habitat modification. For example, increased soil nutrients may play a role in seedling performance following bamboo flowering, as in temperate forests dominated by *Sasa kurilensis* in Japan (Abe et al., 2001, 2002) and by *Bashania fangiana* in China (Taylor and Qin, 1988). Such resource change is at the heart of the intraspecific competition hypothesis (Janzen, 1976; Gadgil and Prasad, 1984; Taylor et al., 1991; Franklin and Bowman, 2003), which explains the long clonal phase of bamboos as habitat modification. Stearns (1980) habitat modification theory suggests delayed flowering is selected for when adult longevity changes environmental conditions such that successful seedling recruitment is increased. Bamboo expands vegetatively over a long period of time sequestering a large spatial patch, then flowers and dies, leaving ample resources and little competition in its wake for seedlings to germinate and grow. In addition, the sheer size of the genet may also play a role. The longer the clone waits, the larger the genet, and larger genets tend to produce more seed (Matsuo et al., 2014).

Another mechanism of delayed reproduction may be seed predation (e.g., rodent outbreaks following flowering; Jaksic and Lima, 2003), the so-called predator satiation hypothesis (Janzen, 1976; Gadgil and Prasad, 1984). Based on this hypothesis, a higher seedling establishment probability would be expected during gregarious flowering events because not all seeds are consumed. Kakishima et al. (2011) found evidence for selection of a 6-year semelparous cycle of *Strobilanthes flexicaulis* via pollinator activity and predator satiation; i.e., pollination was more likely and seed predation less likely when gregarious flowering occurred. Kitzberger et al. (2007) also found lower *Chusquea culeou* seed predation rates in flowered areas versus those that had not flowered, as did Abe et al. (2001, 2002) for *S. kurilensis* and Caccia et al. (2015) for *C. culeou*. One argument against the predator satiation hypothesis is that there is no clear selection force that would result in such long periods (i.e., multiple decades) of clonal growth (Keeley and Bond, 1999). In addition, there is mounting evidence of small-scale flowering events of bamboo species (Miyazaki et al., 2009; Kitamura and Kawahara, 2011) that may fail to satiate predators.

A third mechanism put forth to explain long vegetative periods prior to flowering is the resource hypothesis (Gadgil and Bossert, 1970), which suggests that a period of time is needed to acquire the necessary resources for flowering, and that the resources are exhausted during the flowering event. The latter explains the death of culms and how plants sacrifice growth for the sake of reproduction (Abrahamson and Caswell, 1982). The energy allocated to reproduction gradually increases as buds become flowers, flowers are fertilized and seeds mature (Harper and Ogden, 1970). Lupine [*Lupinus nanus* ssp. *latifolius* (Benth.) D. Dunn.] distributes up to 61% of its energy to reproductive tissues and 29% to seeds (Pitelka, 1977), but such studies are lacking for bamboo. One study shed a bit of light. Miyazaki et al. (2009) was able to show that non-flowering culms transferred carbon to flowering culms, suggesting an energy need was being met through physiological integration of the clonal bamboo. The increase of energy to reproductive organs may thus decrease energy allocated to vegetative organs, and it is reasonable to predict that bamboo may allocate a large proportion of energy to reproduction during flowering that leads to culm death.

The success of seedling establishment and growth relies on both biotic (birds, mammals, insects, etc.) and abiotic (light, water, nutrients, etc.) factors (Grubb, 1977; Harper, 1977; Grime, 1979). Several studies have highlighted the effects of flowering on both bamboo seedlings and heterospecific tree species. For some bamboo species, restoration of bamboo forest relies exclusively on regeneration from seeds after bamboo flowers and dies, because rhizomes die after culms flower (Tian, 1987; Keeley and Bond, 1999); although, this may not include the entire genet (Miyazaki et al., 2009). Whether large-scale gregarious flowering or small-scale events, bamboo regeneration is clearly greatest in the gaps left behind by flowering and subsequent death of culms (Marchesini et al., 2009).

The positive response of bamboo seedlings to flowering seems mainly driven by an increase in light to the forest floor following die-off of culms. For example, *S. kurilensis* and *C. ramosissima* seedling growth was positively related to increased light levels but showed little response to other environmental variables (Makita, 1992; Montti et al., 2011). Makita (1992) described the post-flowering regeneration response as three phases: establishment, density-stable, and thinning, where ramets eventually die due to competition with neighboring ramets.

The flowering of arrow bamboo *F. qinlingensis*, one of giant panda’s main food resources in the Qinling Mountains, offers a valuable opportunity to examine delayed flowering mechanisms in bamboo. We monitored the flowering event for 5 years, with an analysis of energy availability in flowered and vegetative culms to elucidate exhaustion of resources, and a nutrient addition study to examine habitat modification.

Our objectives were to: (1) document bamboo internal energy resources during flowering, (2) document bamboo regeneration following die-offs, and (3) add evidence toward our understanding of selection for delayed flowering. In reference to hypotheses of delayed flowering, we expected seedlings to grow better in sites that had flowered and died with little advantage from additional fertilization, supporting mostly a habitat modification hypothesis.

## Materials and Methods

### Study Area

**FIGURE 1:**
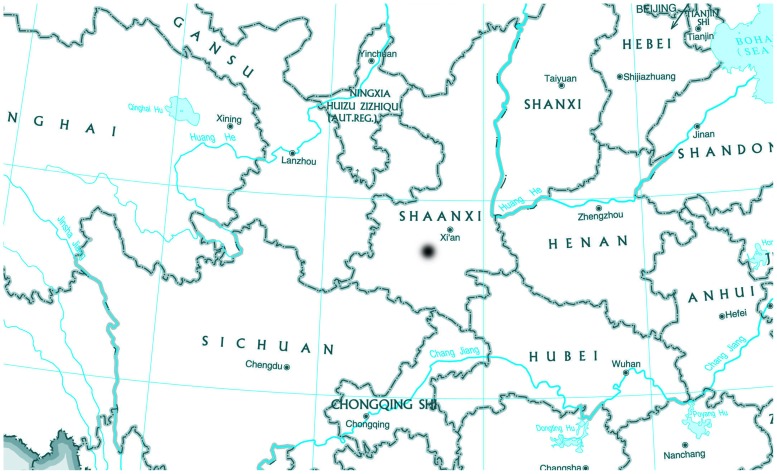
**Study site location**.

This study was carried out at the Taibaishan National Natural Reserve (TNNR, 33° 49′ 30′′ – 34° 05′ 35′′ N, 107° 22′ 25′′ – 107° 51′ 30′′ E) in the Qinling Mountains, Shaanxi Province, China (**Figure 1**). TNNR is in the southern end of the warm temperate zone with four distinguished seasons. It is the northern range of giant panda’s distribution. Mean annual temperature is 8.4°C; – 4.2°C in January and 20.4°C in July. Precipitation averages 945.5 mm/year with 50% falling between July and September. The soil in the flowering area is rhogosol brown soil (pH 6.2) that forms from granite. The lower part of the soil is semi-weathered parent material with pH 6.5.

Vegetation is diverse due to the coexistence of both northern and southern Chinese taxa. Forest communities cover 81% of TNNR, made up of four general vegetation types along an elevation gradient: oak forests (1500–2000 m), birch forests (2000–2500 m), coniferous forests (2500–3540 m), and alpine shrublands and meadows (3450–3700 m). Two main bamboo species are distributed in Qinling Mountains; *F. qinlingensis* predominates at higher elevations (1700–3000 m) and *B. fargesii* (Camus) Kenget Yi at lower elevations (900–1900 m).

### Study Species

Fargesia qinlingensis has a pachymorph rhizome (Li et al., 2003), and its culm can grow up to 3.6 m in height and 13 mm in basal diameter. New shoots regenerate from rhizomes in May and June; during flowering years, flowers appear in April and seeds are set in June. Like most other bamboos in tropical and temperate regions (Janzen, 1976; Gadgil and Prasad, 1984), F. qinlingensis is a perennial monocarpic species which is known for its long period of vegetative growth followed by a mast seeding; it has a seeding cycle of ca. 50 years (Tian, 1987). In TNNR, sporadic flowering of F. qinlingensis began in 1999 in a watershed and mast flowered throughout this watershed in an area of ca. 300 ha from 2001 to 2003 (Yue and Li, personal communication). The temporal gregarious flowering with a few early, a few late, and most flowering during 1 year has been documented in other bamboo species (Abe and Shibata, 2012; de Carvalho et al., 2013). A small amount of un-flowered patches of mature culms, approximately elliptical in shape, grew within a matrix of dead and flowering culms. Similar mosaic patterns of live and dead culms have been reported for several other bamboos (Schaller et al., 1985; Taylor and Qin, 1987, 1988; Johnson et al., 1988; Reid et al., 1989; Taylor et al., 1991; Widmer, 1998; Marchesini et al., 2009; Mizuki et al., 2014).

### Field Methods

Three sites with un-flowered patches were randomly selected for study. Unfortunately, un-flowered patch of site 2 flowered in 2003 and 2004, but only a few sporadic stems (i.e., it does not match the flowering sites we chose). Due to low sample size, we chose to maintain this site as a non-flowering patch and consider our analyses as conservative. The length and width of each patch was measured, and area calculated by using an elliptical formula. The areas of the three un-flowered patches were 240.33, 66.76, and 159.06 m^2^, respectively. The three sites were within a flowering area of about 10 km^2^ with elevation from 1850 to 1930 m, had similar topographic characteristics, canopy cover, tree composition and bamboo cover (**Table 1**). Sites ranged from 1 to 3 km apart. Herb cover was greatest in site 1. All three sites were in a broad-leaved forest mixed with a very small proportion of conifers. There were clearly defined layers of canopy, sub-canopy, and understory. Dominant canopy species consisted of *Quercus baronii* Skan, *Q. aliena* Bl. var. *acuteserrata* Maxim, and *Carpinus turczaninowii* Hance. Species in the shrub layer mainly included *Morus alba* Linn, *Lespedeza bicolor* Turcz, and *Symplocos paniculata* (Thunb) Miq. The understory herbaceous layer was mainly formed by *Oxalis acetosella* Linn, *Hippochaete hiemale* (Linn) Borner, and *Smilax riparia* A. DC, with *F. qinlingensis* dominating.

**Table 1:** The characteristics of three un-flowered patches of *Fargesia qinlingensis* at the Taibaishan National Natural Reserve, Shaanxi Province, China.

Patch	Area (m^2^)	Slope steepness (°)	Slope aspect (°)	Elevation (m)	Canopy cover (%)	Canopy height (m)	Herb cover (%)	Bamboo cover (%)	
								Non-flowering site	Flowering site
1	240.33	26	234	1850	85	18	70	75	45
2	66.76	18	162	1910	80	18	30	70	50
3	159.06	30	180	1880	70	17	25	60	45

#### Habitat Modification Experiment

Six 1 m × 1 m plots were randomly set up in each of the three un-flowered patches. Another adjacent six 1 m × 1 m plots were randomly set up in each flowering area. Plots were at least 5 m apart. In each 1 m × 1 m plot, basal diameter of each culm (i.e., ramet) was measured to its nearest 0.01 mm in October 2003, and re-measured over the next 4 years (2004–2007). Culms were divided into four categories: new shoots, flowering culms, live culms, and dead culms. New shoots (<1 year old) and culms (>1 year old) specifically refer to vegetative reproduction, not seedlings. Seedlings have weak structure and more branches while new shoots are sturdier; seldom have braches, and adjacent to older bamboo shoots.

Total seedlings, all new shoots from seed, of *F. qinlingensis* were counted in each 1 m × 1 m plot from 2004 to 2007. No seedlings were established in 2003 or after 2004 (i.e., seedlings only emerged in 2004). In 2004–2007, ten randomly selected seedlings, or all seedlings if there were less than 10 seedlings in the 1 m × 1 m plot, were measured for height and leaf length to the nearest 0.1 cm.

In the fall of 2006, we performed a fertilization experiment with 1 m × 1 m plots (six per treatment) in a randomized block design with four treatments (+N, +P, N + P, and no fertilizer addition) in each flowering site (sites were blocks). This research examines one aspect of the habitat modification hypothesis: we would expect fertilization to have no effect if the die-off already provides ample resources for seedling recruitment, survival and growth. Measurements of bamboo seedlings (taken at the start and 1 year later in 2007) included density, height, number of branches, number of leaves, and the length of the three longest leaves (subsequently averaged). We calculated plot averages of each variable prior to running analyses, and analyzed the change over the 1 year period.

#### Seed Predation Experiment

Twenty flowering culms were randomly selected from each of the three flowering sites in October 2003; *N* = 60. Florets and actual seeds on each culm were counted. In addition, five 0.5 m × 0.5 m plots were randomly set up in each flowering site. Soil of 10 cm deep in each plot was extracted and all viable seeds (with white embryo) were counted. We also randomly selected and marked 10 culms that started to flower in 2003 in each flowering site. They were revisited in August 2004 and June 2005 to determine survival.

#### Resource Experiment

In October 2003, ten flowering culms from each flowering site and un-flowered patch (*n* = 10; *N* = 60) were randomly collected, sorted into culm parts (stems, branches, and leaves; one sample of each treatment of rhizomes and seeds), and then dried for analysis. Dried culm samples were weighed and randomly split in two subsamples to garner enough material for chemical analyses (two sets of analyses). Chemical constituents in each culm part were analyzed at Mississippi State University, providing dry matter percentage of organic matter (OM), crude protein (CP), neutral detergent fiber (NDF; lignin, cellulose, and hemi-cellulose), acid detergent fiber (ADF; lignin and cellulose), hemi-cellulose (HC = NDF – ADF), and extract ether (EE; crude fat). CP and EE represent energy reserves in the plant while other variables represent structural components.

### Data Analysis

#### Habitat Modification Experiment

We used a repeated measures ANOVA in a randomized complete block design to test the flowering effect (two levels: flowering site and un-flowered patch) and time effect (five levels: 2003, 2004, 2005, 2006, and 2007) on the density of live culms. Plots within patches were averaged and patches considered replicates. Flowering and time were considered fixed effects. If there was a significant interaction between the flowering effect and time effect, a multiple comparison with Bonferroni adjustments was performed. Density of seedlings, and height, number of leaves and average length of longest leaf of individual seedlings were analyzed with a similar repeated measures analysis (using blocks as replicates), except that the time treatment had only four levels (2004–2007). These analyses were performed using the GLM procedure in SAS (SAS Institute, 2013).

For the fertilization experiment, a blocked (by site) MANOVA followed by ANOVAs examined the effects of fertilization on plant growth (change from 2006 to 2007) for stem height, number of branches, number of leaves, leaf length, and seedling density using the GLM procedure in SAS (SAS Institute, 2013).

#### Resource Experiment

A randomized complete block design with two replications in each experimental unit (bamboo culm) was analyzed by using the MIXED procedure in SAS/STAT (SAS Institute, 2013) to test for flowering effect (two levels: flowering site and un-flowered patch) and culm part effect (three levels: leaf, branch, and culm) on the six chemical constituent variables (OM%, CP%, NDF%, ADF%, HC%, and EE%). Two main effects (flowering and culm part) were considered fixed, and block effect was considered random. If there was a significant interaction between the two main effects, a multiple comparison with Bonferroni adjustments was performed to seek significant differences among six treatment combinations (flowering/stem; flowering/leaf; flowering/branch; un-flowered/stem; un-flowered/leaf; un-flowered/branch) for each dependent variable. These analyses were performed using the MIXED procedure in SAS (SAS Institute, 2013). Percentage data were arcsin square root transformed prior to analysis to meet the assumptions of normality and homogeneity of variances. For all analyses, differences were considered significant at *P* < 0.05 level.

## Results

### Habitat Modification Experiment

#### Dynamics of Density and Characteristics of Culms

**FIGURE 2:**
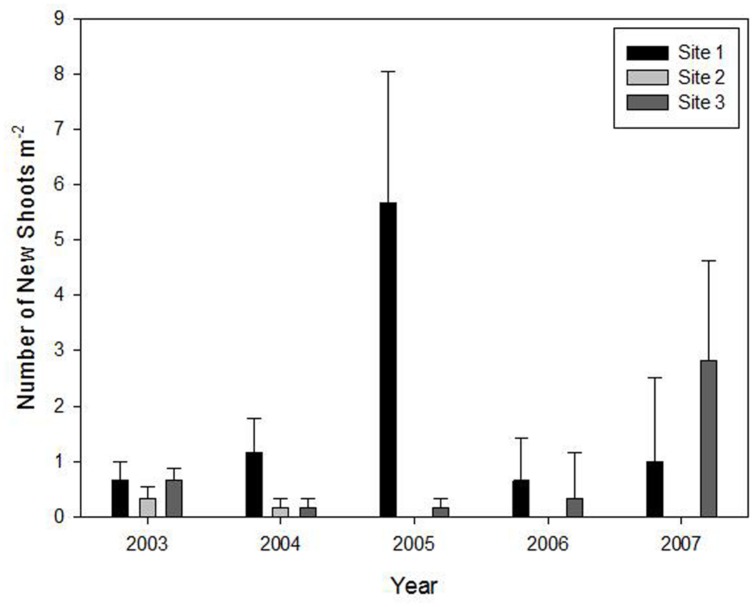
**Means (±1 SE) of density of new shoots of *Fargesia qinlingensis* in the three un-flowered patches (there were no new shoots found in flowering sites) during the 5-year period at the Taibaishan National Natural Reserve, Shaanxi Province, China**.

No new shoots regenerated from rhizomes in the flowering sites during the five studied years. However, new shoot regeneration occurred every year in the three un-flowered patches except site 2 that only flowered in 2003 and 2004 (**Figure 2**).

**(A)** Number of live culms, **(B)** flowering culms, **(C)** live culm biomass.

**FIGURE 3:**
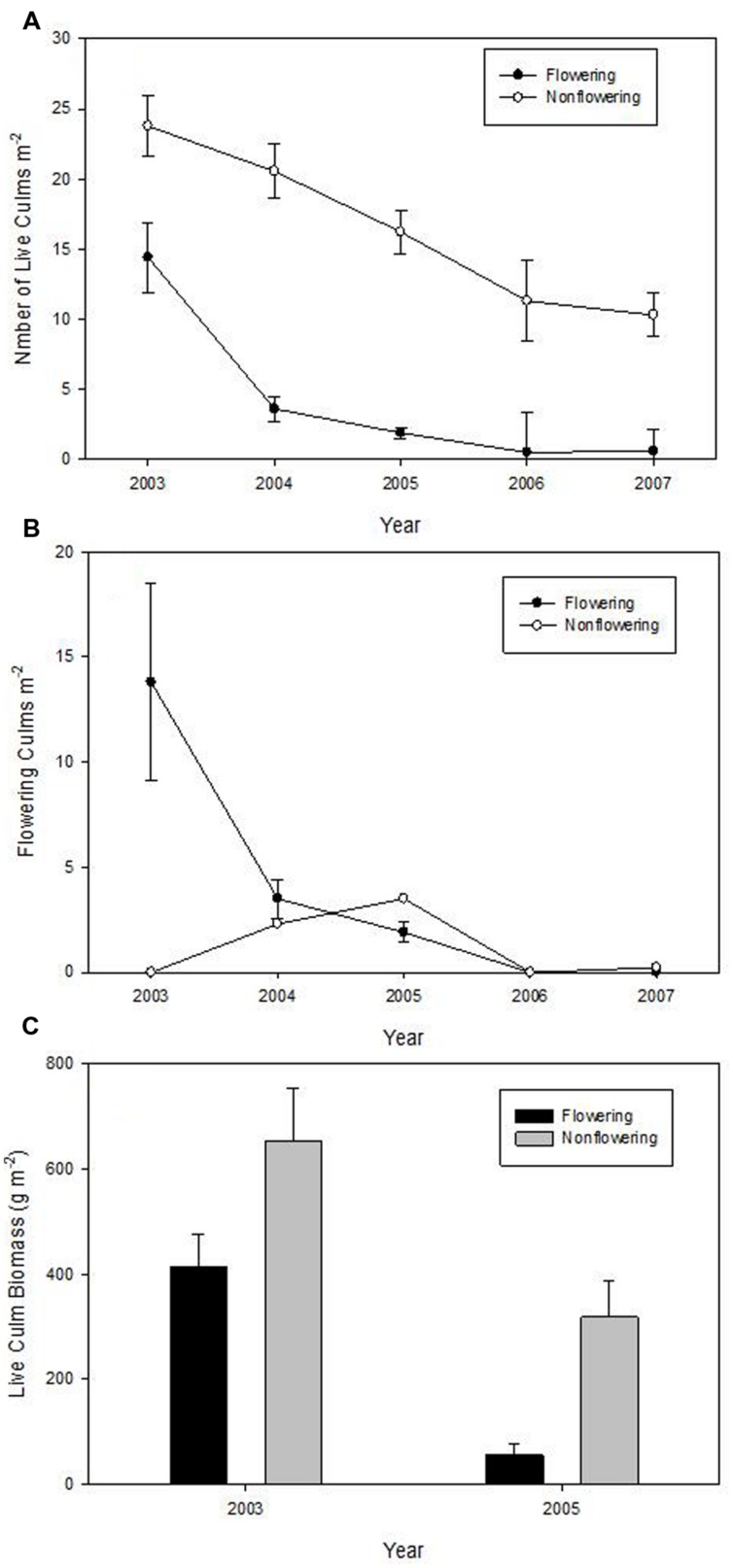
**Means (±1 SE) of the density of live culms of *Fargesia qinlingensis* in the flowering sites and un-flowered patches over 5-year period at the Taibaishan National Natural Reserve, Shaanxi Province, China. (A)** Number of live culms, **(B)** flowering culms, **(C)** live culm biomass.

There was a significant effect of flowering on the density of live culms (*F* = 14.09, *P* = 0.0199), but no time (*F* = 26.71; *p* > *F* = 0.1440) or time^∗^treatment effect (*F* = 36.91; *p* > *F* = 0.1228). Density decreased dramatically in the flowering site over the 5 years, but surprisingly, density of live culms in un-flowered patches also significantly decreased (**Figure 3A**). The density of live culms in un-flowered patches was significantly greater than that in flowering sites every year (**Figure 3A**). Four of 30 culms that started to flower in 2003 died in 2004; half of them (15 culms) were still alive in 2005, but none remained in 2006.

Number of flowering culms decreased from about 14/m^2^ to zero within 5 years in the flowering sites, while un-flowered patches had limited flowering (<5 culms/m^2^) in both 2004 and 2005 (**Figure 3B**). Live culm biomass decreased by about 86% in the flowering patches but only by about 52% in the un-flowered patches (**Figure 3C**).

#### Density and Characteristics of Seedlings

**FIGURE 4:**
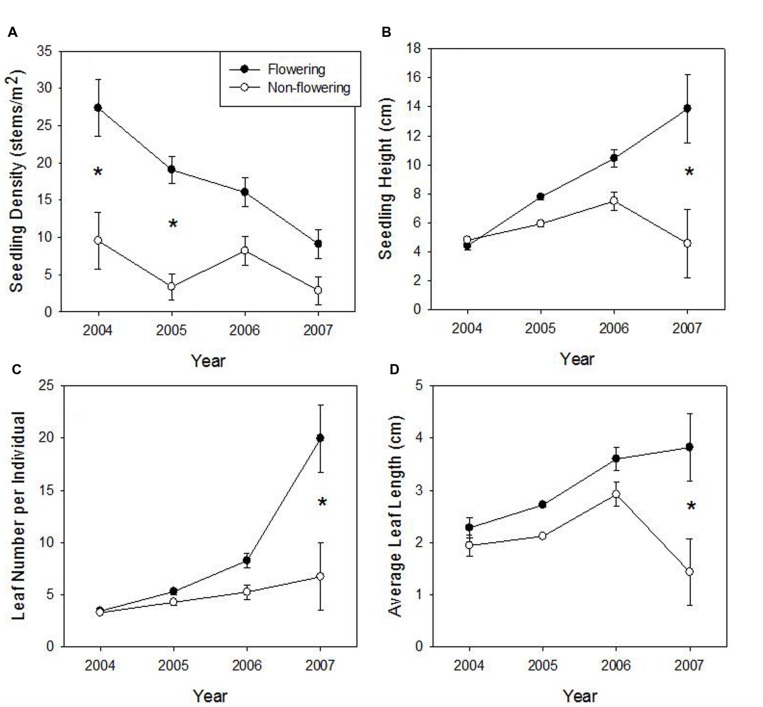
**Means (±1 SE) of density of seedlings **(A)** seedling height **(B)**, seedling leaf number **(C)**, and average seedling leaf length **(D)** at the Taibaishan National Natural Reserve, Shaanxi Province, China.** Asterisks indicate significant difference in flowering and un-flowered sites for a particular year at *P* < 0.05 level with Bonferonni correction.

No seedlings were established until 2004. We found no significant effect of time and flowering interaction on either seedling density or average leaf length (**Table 2**). Both variables had a significant effect (greater in flowering areas; **Figures 4A,D**) and seedling density also decreased significantly over time (**Figure 4A**). Both seedling height (**Figure 4B**) and leaf number (**Figure 4C**) had a significant time^∗^flowering effect, in that flowering and non-flowering areas showed a similar increase until the last time period when flowering site characteristics clearly diverged to being significantly greater (a trend also shown with average longest leaf). Survivorship from 2004 to 2007 was 32 and 40% in flowering and un-flowered patches, respectively.

**Table 2:** Repeated analysis of density, height, leaf number, and average leaf length of seedlings of *Fargesia qinlingensis* at the Taibaishan National Natural Reserve, Shaanxi Province, China.

Fixed Effects	Seedling Density		Seedling Height		Leaf Number		Leaf Length	
	*F*	*P*	*F*	*P*	*F*	*P*	*F*	*P*
Flowering (F)	34.31	0.0042	27.52	0.0345	14.22	0.0637	35.26	0.0272
Time (T)	9.44	0.0018	5.28	0.0404	14.08	0.0040	3.16	0.1070
T × F	2.93	0.0769	4.93	0.0465	6.62	0.0248	2.94	0.1209

*Variable flowering had two levels: flowering sites and non-flowering patches. Variable time had four levels: 1–4 years old.*

#### Fertilization

**FIGURE 5:**
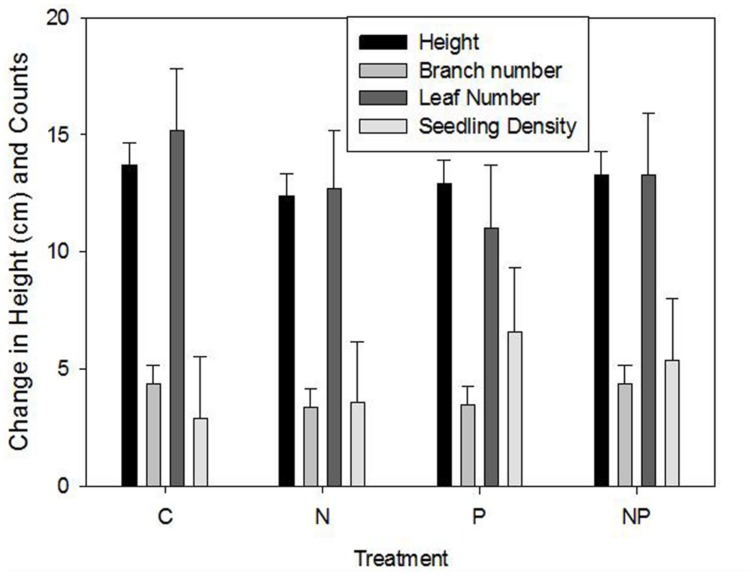
**Mean (±1 SE) height, branch number, leaf number, and seedling density for control (C), nitrate addition (N), phosphorus addition (P), and combined N + P addition.** There were no significant differences.

No significant effects were found among fertilization treatments for change (growth between 2006 and 2007) in stem height (*F* = 0.34, *p* > *F* = 0.7964), number of branches (*F* = 0.52, *p* > *F* = 0.6678), number of leaves (*F* = 0.45, *p* > *F* = 0.7177), leaf length (*F* = 0.69, *p* > *F* = 0.5603), or seedling density (*F* = 0.41; *p* > *F* = 0.7485). Height growth increased by approximately 13 cm, number of branches and number of leaves increased by 4 and 12, respectively, and leaf length increased by 0.7 cm (**Figure 5**). Interestingly, ramet density also increased, averaging as little as 2.9 individuals m^–2^in control plots, to 3.6, 5.4, and 6.6 in N, NP, and P plots, respectively.

### Seed Predation Experiment

#### Seed Density and Seedling Establishment

In the three flowering sites, average floret density (2687 ± 847 florets m^–2^) was much greater than average density of actual seeds on culms (53 ± 20 seeds m^–2^) and seeds in the soil seed bank (5 ± 2 seeds m^–2^). Density of 1-year-old seedlings averaged 27 ± 2 seedlings m^–2^ (**Table 3**).

**Table 3:** Flower and seed characteristics of *Fargesia qinlingensis* from three flowering sites in the Taibaishan National Natural Reserve, Shaanxi Province, China, in October 2003.

Site	Total florets / culm (±1 SE)	Actual seeds/ culm (±1 SE)	Florets/m^2^ (±1 SE)	Actual seeds on culms/m^2^ (±1 SE)	Seeds in soil/m^2^ (±1 SE)	Seedlings/m^2^ (±1 SE)
1	183.5 (30.4)	3.8 (1.5)	3303 (843.6)	68.4 (17.5)	8.8 (2.9)	29.8 (5.2)
2	299.6 (39.9)	6.2 (1.6)	3745 (253.6)	77.5 (5.25)	5.2 (2.3)	28.7 (13.2)
3	242.9 (44.9)	3.0 (0.8)	1012.1 (211.9)	12.5 (2.6)	1.8 (0.8)	23.6 (9.6)
Average	242.0 (33.5)	4.3 (1.0)	2686.7 (847.0)	52.8 (20.3)	5.3 (2.0)	27.4 (1.9)

### Resource Experiment

**FIGURE 6:**
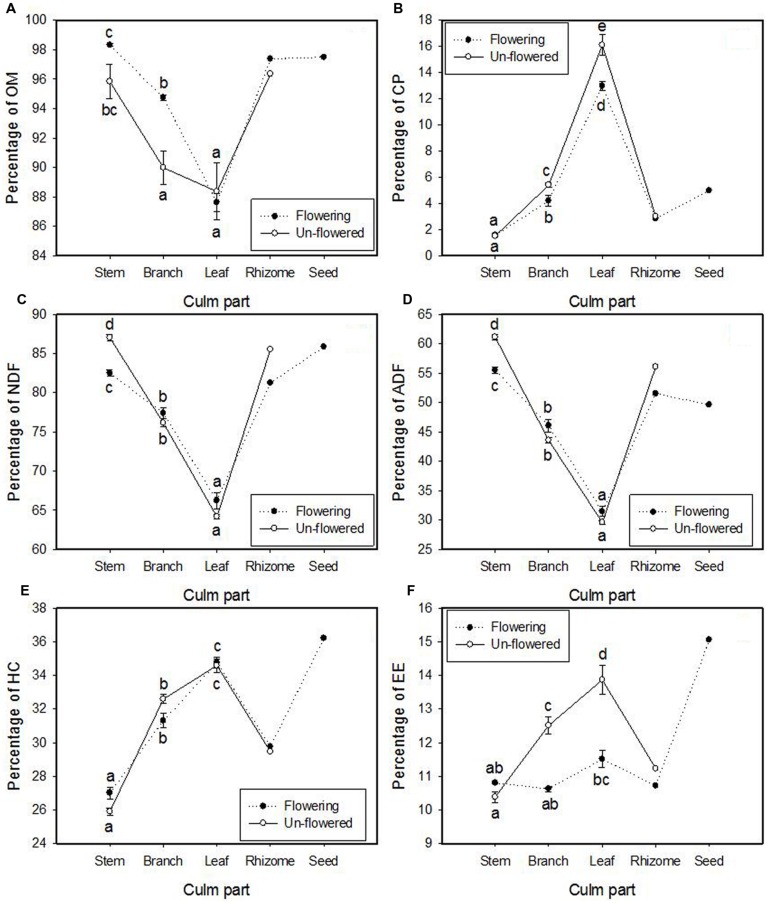
**The means (±1 SE) of treatment combinations for seven chemical compound variables: **(A)** organic matter (OM), **(B)** crude protein (CP), **(C)** neutral detergent fiber (NDF; lignin, cellulose, and hemi-cellulose), **(D)** acid detergent fiber (ADF) (lignin and cellulose), **(E)** hemi-cellulose (HC), and **(F)** ether extract (EE; crude fat) in culms of *Fargesia qinlingensis* collected from the Taibaishan National Natural Reserve, Shaanxi Province, China.** Different letters indicate significant differences at *P* < 0.05 level. Two samples of rhizome (flowering and un-flowered) and one sample of seeds were also graphed for comparison but not included in analyses.

The interaction of flowering and culm part type was significant for all six chemical compounds (**Table 4**). OM% was significantly greater in flowering branches than in un-flowered branches (**Figure 6A**). Leaves contained much more CP% than branches and stems (16.1 and 13.0% compared to 5.4 and 4.2% in branch and 1.5 and 1.6% in stem for un-flowered culms and flowering culms, respectively), and CP% in leaves and branches was significantly greater for un-flowered culms than for flowering culms (**Figure 6B**). Two less-digestible fiber constituents (NDF% and ADF%) had the same trend: stems > branches > leaves. Fibers’ percentages in stems were significantly greater for un-flowered culms than flowering culms, but the differences were not significant for branches and leaves (**Figures 6C,D**). Hemi-cellulose (HC%) was significantly different among culm parts: leaves > branches > stems, but there was no significant difference between flowering culms and un-flowered culms (**Figure 6E**). Leaves contained more crude fat (EE%) than branches and stems for un-flowered culms (13.9% compared to 12.5 and 10.4% in branches and stems), and crude fat in leaves and branches was significantly greater for un-flowered culms than flowering culms (**Figure 6F**).

**Table 4:** Summary of six mixed linear models for chemical compound variable of *Fargesia qinlingensis* collected from the Taibaishan National Natural Reserve, Shaanxi Province, China.

Fixed effects	*F* value					
	OM%	CP%	NDF%	ADF%	HC%	EE%
Flowering (F)	6.44_(1,2)_	15.04_(1,2)_	2.96_(1,2)_	0.47_(1,2)_	0.00_(1,2)_	23.28_(1,2)_^∗^
Part (P)	37.32_(2,26)_^∗∗∗^	1178.81_(2,26)_^∗∗∗^	670.52_(2,26)_^∗∗∗^	1019.37_(2,26)_^∗∗∗^	346.81_(2,26)_^∗∗∗^	48.53_(2,26)_^∗∗∗^
F × P	3.64_(2,26)_^∗^	9.11_(2,26)_^∗∗^	28.04_(2,26)_^∗∗∗^	27.25_(2,26)_^∗∗∗^	7.33_(2,26)_^∗∗^	24.33_(2,26)_^∗∗∗^

*OM, organic matter; CP, crude protein; NDF, neutral detergent fiber (ligning, cellulose, and hemi-cellulose); ADF, acid detergent fiber (ADF; lignin and cellulose); HC, hemi-cellulose (HC = NDF – ADF); and EE, extract ether (crude fat). Variable Flowering had two levels: flowered site and un-flowered site. Variable Part had three levels: leaf, branch, and culm. ^∗^P < 0.05, ^∗∗^P < 0.01, ^∗∗∗^P < 0.001. Numbers in parenthesis represent numerator and denominator df.*

Two rhizomes from a flowering site and an adjacent un-flowered patch were analyzed, and seeds from several flowering culms were also analyzed, rendering only enough material for one set of chemical constituent analyses each (*n* = 1). Greater amount of easily digestible crude fat and partially digestible hemi-cellulose were found in seeds compared to all other culm parts (**Figures 6E,F**). Chemical amounts in rhizomes were similar to branches and stems. Little difference was found between the flowered and un-flowered rhizome samples, but flowered rhizomes generally had lower chemical concentrations.

## Discussion

Understanding the dynamics of gregarious flowering events and their impacts on ecosystem structure and community dynamics is imperative for conservation management. In addition, the evolutionary basis of delayed and synchronized flowering is still debated and data are needed to compare contrasting views (Saha and Howe, 2001). We first answer general questions about the timing and structural changes during a *F. qinlingensis* flowering event and then interpret data in relation to hypotheses of delayed reproduction.

### Flowering, Semelparity, and Reproduction

We first wanted to determine if flowering was gregarious. Flowering started in 1999 in the area, as documented by locals, but the peak flowering year was 2003 – word spread about the event which is what prompted us to begin our study. Our data documents much lower flowering occurrence since 2003. Thus, the gregarious flowering event can be described more as an asymptote in time rather than a specific year, as has also been suggested by several other studies (Veblen, 1982; Gadgil and Prasad, 1984; Banik, 1995; Widmer, 1998; Kitamura and Kawahara, 2011) and is perhaps more typical than large-scale gregarious events for temperate bamboo species (Makita, 1998; Keeley and Bond, 1999).

We also wanted to document the semelparity of *F. qinlingensis*. We found that half of the flowering culms of *F. qinlingensis* survived for at least 2 years. A similar phenomenon has been reported for individuals of *S. veitchii* var. *hirsute* (Abe and Shibata, 2014) and *F. robusta* that lived for 2–3 years after flowering in the Min Mountains (Qin et al., 1993). In addition, new vegetative shoots stopped generating in flowering sites, while in the nearby un-flowered patches, new shoots continued to regenerate every year, so rhizomes and roots had apparently died as well after flowering; thus, *F. qinlingensis* appears semelparous at least in relation to one flowering event. However, since we did not collect genetic data, we are unsure if we have genets flowering and dying or portions of genets, as shown with *Sasa* (Miyazaki et al., 2009), flowering and dying. For *Sasa*, non-flowering culms did not die and thus the genet was not semelparous. This remains to be tested for *F. qinlingensis*.

Our next goal was to document the timing of regeneration. Although flowering started from 1999, no seedlings were established until 2004, suggesting either delayed germination or much higher pollination success (greater number of seeds that escape predation) following the gregarious flowering peak in 2003 when we started data collection. No seedling establishment may be also due to seed limitation (no seed from rather poor seed production before 2003 arrived at the plots); predation or damage of the seeds (for instance by fungi) should also be contributory. Seeds of *B. fangiana* are reported to remain dormant for at least 2 years in the Qionglai Mountains (Taylor and Qin, 1988). Qin (1985) also reports that seeds of *F. scabrida* remain dormant in the soil up to 5 years in the Min Mountains. Recent work on *Chusquea* (González et al., 2002) and *Sasa* (Kitamura and Kawahara, 2011) also identified multi-year dormancy. However, seeds of *F. qinlingensis* showed no dormancy characteristics in the lab, germinating any time of year under both light and dark conditions (Wang et al., 2007). The second alternative, that pollination success is much greater during the peak of gregarious flowering events, is also supported in the literature (Widmer, 1998; Kakishima et al., 2011). Measurements of cross-fertilization success recorded during non-flowering years (at least no gregarious flowering, only local individuals) were much lower (<7%) in *Sasa* species (Mizuki et al., 2014). Regardless of the driver, our data suggest masting promoted seed production and seedling success, as also shown by Abe and Shibata (2014).

Seedlings grew slow in this study. Three-year-old seedlings had heights of 10.5 cm and 7.5 cm in flowering sites and un-flowered patches, respectively. Allelopathy and micronutrients might be considered as a mechanism slowing down regeneration in non-flowering sites, but support for these mechanisms is lacking. Slow growth also was noted for *C. tomentosa* (Stern et al., 1999). Qin et al. (1989) report that 3-year-old seedlings of *B. fangiana* averaged only 6.7 cm high. Reid et al. (1989) report that *B. fangiana* 4-year-old seedlings were no more than 10-15 cm tall, matching 4-year-old seedlings in flowering sites of this study (14 cm). Researchers have estimated the time for bamboo seedlings to grow back to their original full size after a flowering event: 19 years for *F. qinlingensis* in the Qinling Mountains, Shaanxi, China (Tian, 1991); 15–20 years for *B. fangiana* and *F. scabrida* in the Qionglai Mountains, Sichuan, China (Qin et al., 1989; Taylor and Qin, 1993); 20 years for *S. kurilensis* in the Hakkoda Mountains of northern Japan (Makita, 1992); and 16–19 years for *F. denudata* in the Min Mountains, Gansu, China (Huang, 1994). Data comparing *F. qinlingensis* areas that flowered in the early 1980s versus adjacent un-flowered areas (Wang et al. unpublished data) show little difference in density or culm diameter, suggesting the recovery occurs within 20 years following flowering in the Qinling Mountains. These remarkably similar estimates of recovery for temperate bamboo species may be used for management of bamboo forests.

### Support for Hypotheses of Delayed Flowering

We expected seedlings to grow better in sites that had flowered and died with little advantage from additional fertilization. Our results show that flowering of *F. qinlingensis* significantly altered stand characteristics. Live culms dramatically declined in flowering areas from 2003 to 2007, and only a small amount of flowering culms remained in 2005. Surprisingly, live culms also decreased over the 5-year period in the un-flowered patches. One possible explanation is that some culms generate from rhizomes running from flowering areas, and indeed, some of the culms in un-flowered patches did flower in 2004 and 2005. Such small-scale spatial structure in flowering patterns have been shown in other bamboo species with genet-level flowering periodicity; i.e., different genets flower at different times (Mizuki et al., 2014) and even ramets within genets flower at different times (Miyazaki et al., 2009). A second explanation is a changed environment after culms die that affects neighboring un-flowered patches, such as increased light (Taylor and Qin, 1988), temperature fluctuations (Abe et al., 2001, 2002), and predator outbreaks (Jaksic and Lima, 2003).

Seedlings performed better in flowering sites than in un-flowered patches, but as expected the addition of nitrogen and phosphorus had no significant effects. Thus, the better growth of seedlings in flowering sites than in un-flowered patches likely resulted from the resource change, albeit we do not have resource change data, caused by death of culms and a saturation of resources in flowering sites (Taylor and Qin, 1988; Abe et al., 2001, 2002). Greater light levels especially (Makita, 1992; Marchesini et al., 2009) and reduction of mineral nutrient uptake from soils following rhizome death resulted in increased resources that could be used by bamboo seedlings in flowering sites (Taylor et al., 1991). Marchesini et al. (2009) also found higher survivorship of bamboo seedlings in senescent (after flowering) versus live sites. The better performance of seedlings in flowering sites suggests intraspecific competition (Janzen, 1976; Gadgil and Prasad, 1984; Taylor et al., 1991; Franklin and Bowman, 2003); few plants establish and survive beneath dense culms, because most resources for plant growth are used by established culms. Contrarily, Li et al. (2013) found the opposite trend for two *Fargesia* species, where dead culm litter apparently blocked seedling regeneration success. Thus, our data support the habitat modification hypothesis more so than other hypotheses.

We expected to find an exhaustion of resources in flowering culms that would support the resource allocation hypothesis. It has been shown that many plants sacrifice growth for the sake of reproduction (Abrahamson and Caswell, 1982; Iler and Inouye, 2013). The energy allocated to reproduction gradually increases as buds become flowers, flowers are fertilized and seeds mature (Harper and Ogden, 1970); and hence, allocation to vegetative organs decreases. We examine this hypothesis with the knowledge of vegetative propagation and allocation of energy within ramets. No new shoots were found in flowering areas even though culms were still alive. The reason why vegetative reproduction stopped when the sexual reproduction started is probably because of the reallocation of energy from culms to reproduction organs and subsequent death of culms; thus, allocation of all energy was to reproductive stems of *F. qinlingensis.*

Chemical constituents’ allocation in different culm parts of *F. qinlingensis* were similar to other bamboos. Leaves of both flowering culms and un-flowered culms contained more CP than branches and stems. Similar results were reported in *Phyllostachys aureosulcata* McClure by Dierenfeld et al. (1982) and in *B. fangiana* by Qin et al. (1993). But CP% in leaves of these three bamboos is slightly different: *B. fangiana* (19.44%) > *F. qinlingensis* (16.1%, this study) > *P. aureosulcata* (13%). Two fibers’ (NDF and ADF) allocation in *F. qinlingensis* had the same trend: stems > branches > leaves, and matching that of *F. scabrida* (Schaller et al., 1990). Organic matter and hemi-cellulose also had the same trend (leaves > branches > stems) as *F. scabrida* (Schaller et al., 1990). Leaves of un-flowered culms had more crude fat than stems and branches, which is similar to *B. fangiana* (Qin et al., 1993), but crude fat content was much greater in *F. qinlingensis* (10.4, 12.5, and 13.9% in stems, branches, and leaves, respectively) than in *B. fangiana* (0.59, 2.4, and 3.4% in stems, branches, and leaves, respectively).

Organic matter in branches was greater in flowering culms than in un-flowered culms, while less-digestible fibers (NDF and ADF) in stems were greater in un-flowered culms than in flowered culms. CP and crude fat in leaves and branches were greater in un-flowered culms than in flowering culms. One explanation is that the allocation of energy to reproduction decreased energy allocated to vegetative modules as noted above (Leopold and Kriedemann, 1975). Crude fat had the greatest concentration in seeds, and both crude fat and CP were significantly less in branches and leaves of flowering plants, suggesting its reallocation. Albeit not extreme, significant differences (specifically crude fat and CP) always showed flowering culm amounts less than non-flowering culms and support a reallocation for reproduction. However, such energy allocation is true for iteroparous species as well, where there is not a complete exhaustion of resources that the hypothesis suggests. Our results corroborate such allocation based on lower percentages of especially CP and fat, but not complete exhaustion; thus, we do not consider our results as strong evidence for the resource allocation hypothesis.

Finally, we expected to find significant loss of seeds that would support the predator satiation hypothesis (Janzen, 1976). The number of seeds in the soil seed bank and in the culms was much less than the florets, suggesting that a large portion of seeds had been removed or eaten. Although most bamboos set large quantities of seeds (Janzen, 1976), seeds are preyed upon heavily by invertebrates (e.g., insects, Taylor and Qin, 1988; Zheng, 1994) and vertebrates (e.g., rodents and birds, Janzen, 1976). Therefore, our data on seed numbers may underestimate fecundity of *F. qinlingensis*, because rodent outbreaks often follow bamboo flowering (Gallardo and Mercado, 1999; Jaksic and Lima, 2003). We unfortunately do not know how many viable seeds were developed, but we cannot exclude the predator satiation hypothesis as a mechanism for delayed reproduction in *F. qinlingensis*.

Of course, these above hypotheses of delayed reproduction are not mutually exclusive as can be seen based on our predictions (**Table 5**). For example, by default of an exhaustion of resources, adult culms die and create the same patch for seedling reproduction as the habitat modification hypothesis. We found evidence for both hypotheses with further study: no effect of N and P fertilization supported the habitat modification theory and allocation of energy to reproduction supported the resource allocation theory. Of course, resource allocation can also occur in iteroparous species and the modest differences in energy between flowering and un-flowered culms do not strongly suggest an exhaustion of resources. Further, the apparently high loss of seeds supports the predator satiation hypothesis, although none of the other results suggest predator satiation as the main driver of delayed flowering. Taken together, the data most strongly support, and in fact never truly negate, the habitat modification hypothesis.

**Table 5:** Predicted changes based on data collected from a *Fargesia qinlingensis* flowering event at the Taibaishan National Natural Reserve, Shaanxi Province, China, that would support hypotheses regarding delayed flowering.

Hypothesis	Flowering Culms	Seedling Survival	Seedling Growth	Culm Energetics	Vegetative Reproduction	+N, +P	Reproduction
Habitat Modification	Die	F > NF	F > NF	F≈NF	F = NF	**No effect**	Seed potential≈seed persistence
Resource	Die	F > NF	F > NF	**F < NF**	**F < NF**	Increased growth	Seed potential≈seed persistence
Predator Satiation	**Survive**	**F = NF**	**F = NF**	F≈NF	F = NF	Increased growth	**Seed potential > seed persistence**

*Predicted changes in bold represent unique responses for one hypothesis. ^∗^F indicates Flowering, and NF indicates Non-Flowering. If the main driver of delayed flowering follows the Habitat Modification hypothesis, the habitat after flowering should be more favorable (ample resources and little competition) for seedling survival and growth (F > NF), but we should not find significant effects on culm energetics, vegetative reproduction, and fertilizer addition. If the main driver of delayed flowering follows the Resource hypothesis, culm energetics and vegetative reproduction of Flowering should be less than that of Non-Flowering because all resources of the former will be used by flowering ramets. If the Predator Satiation hypothesis is the main driver of delayed flowering, the seed potential would be greater than persistence in Flowering areas but approximately equal in Non-Flowering areas while the performance of seedlings, culm energetics and vegetative reproduction should not be different.*

## Conclusion

The characteristics of bamboos and bamboo stands were dramatically altered during this flowering event in terms of culm dynamics, seedling establishment, and chemical constituent allocation. We have shown evidence that allocation of energy toward growth is sacrificed for allocation toward flowering and fruiting, but the amount does not appear dramatic enough to fully support an exhaustion of resources hypothesis. Our data suggest slow seedling growth, enhanced growth in areas without a bamboo canopy, and a long regeneration time for bamboo stands, supporting the habitat modification hypothesis for semelparity. We have conflicting results regarding the predator satiation hypothesis. Seed dormancy would seem to argue against such a hypothesis, while the low number of regenerating individuals compared to the number of potential seeds from counted empty florets, and their being distributed into un-flowered areas, suggests predation was a factor. While we have no data on seed herbivory, we agree with Keeley and Bond (1999) that suggest delayed flowering for extended periods (c. 50 years for *F. qinlingensis*) seems too long for the sole purpose of off-setting herbivore populations. We also suggest that multiple forcing factors may have led to the development of delayed flowering, and that the more recent evidence that larger genets are more productive (Matsuo et al., 2014) should be added as a hypothesis.

## Author Contributions

WW: helped conceive research ideas, helped collect field data, helped analyze data, and write manuscript. ZL: helped conceive research ideas, helped collect field data, helped analyze data, and write manuscript. SF: helped conceived research ideas, helped with analysis, and writing manuscript. BR: helped conceive research ideas, performed all analysis of tissue energy, and edited manuscript.

## Conflict of Interest Statement

The authors declare that the research was conducted in the absence of any commercial or financial relationships that could be construed as a potential conflict of interest.
